# Shock temporality: international students coping with disrupted lives and suspended futures

**DOI:** 10.1007/s12564-022-09793-2

**Published:** 2022-08-31

**Authors:** Catherine Gomes

**Affiliations:** RMIT University, Melbourne, Australia

**Keywords:** International students, Research students, Australia, Transience, Temporality/temporalities, Pandemic

## Abstract

During the COVID-19 pandemic, people around the word experienced periods of local, state and international immobility due to lockdowns, border closers and travel restrictions. For transient migrants such as international students, these kinds of immobility have resulted in disrupted lives with professional and personal futures suspended as careers and relationships become stuck in limbo. Moreover, such sudden and extended periods of immobility have not been sufficiently covered in temporality literature due to the novelty of the pandemic crisis in the international education, migration and mobility studies spaces. By conducting a pilot project investigating current and recently graduated higher degree by research (HDR) international students (PhD, Masters and Honours) from public institutions in the Australian city of Melbourne, this paper, thus, introduces the concept of ‘shock temporality’ caused by the global COVID-19 pandemic. Shock temporality takes place when the expected and finite temporary/transitory experience becomes forcefully broken and appears to be ongoing outside of the individual’s control. While shock temporality has left HDR international students’ professional and personal agendas and aspirations in suspension, students interviewed use the time to plan and prepare for truncated futures. The findings of this paper, thus, become relevant in assisting higher education student support services in creating potential approaches and strategies for a post-pandemic future.

## Introduction

This paper reports on the findings of a pilot study conducted in 2021 – during the height of COVID-19 restrictions in the Melbourne city of Australia. The aim of the study was to understand the lived experiences of current and recently graduated higher degree by research (HDR) international students (PhD, Masters and Honours)^1^ in order to assist higher education student support services with early (post)pandemic wellbeing recommendations. At the time of writing, the higher education sector in Australia was facing mass voluntary and forced redundancies across academic and professional (administration) staff lines due to the drop in international students because of closed international borders. Higher education institutions such as the Group of Eight (G8) research-intensive public universities worried about conducting reseach since they used international student fees to fund their research (Australian Bureau of Statistics, 2020). Since 29 per cent of university students in Australia have been international students in pre-pandemic years (Universities Australia, 2020), lower numbers of these students led to questions of institutional sustainability. Hence, the result was the discontinuation of courses and the restructuring or phasing out of academic departments and schools.^2^

The premise of this paper is that the international student experience is not limited to the course experience – quality of life is equally important (Forbes-Mewett, 2019; Marginson, 2014; Tran & Dempsey, 2017). Research has shown that returning to pre-pandemic student wellbeing practices and policies which made little distinction between domestic and international student needs will not work because of:

- risk averse students choosing to remain in the home country due to ongoing uncertainties around government and community responses to the pandemic;
- the increased visibility and prestige of home institutions which have had the opportunity to attract students who would normally have travelled overseas to Western institutions for their education;
- the perception that overseas institutions failed to meet their duty of care towards international students during the pandemic as interviews with international students in media reports suggest;
- Mainland Chinese media in conjunction with its government discouraging (future) students from studying in Australia due to increasing geopolitical tensions; and
- the continuation of the virtualisation of higher education (Hurley, 2020, Zhang 2020).

Lived experience research on international students as transient migrants is therefore essential and urgently required in order to underpin the recovery of the international education sector by providing insights informing practice pertaining to student wellbeing and support.

Before the COVID-19 global pandemic, there was increasing scholarship calling for a more holistic understanding of the international student experience which is not solely steeped in the study experience but includes the non-study lived experiences of students (Gomes, 2022, Phan et al., 2019). These scholars suggest that the lived experiences of international students—including accessibility to goods and services, employment and employability, self-perceived identities, social and cultural cohesion, aspirations and media/communication use—directly correlate with the ways students engage with their courses. Rapidly emerging academic commentary in 2020 on the impact of the global pandemic on international students in Australia and elsewhere has concentrated specifically on economic and financial losses (Hurley, 2020), international student mobilities (ISM) (Yang, 2022**,** Van de Velde et al., 2021, Bardill et al. 2021, Sustarsic & Zhang, 2021, Sidhu et al., 2021) international student future enrolment and mobility (Qi & Ma, 2021), student rights and wellbeing (Farbenblum & Berg, 2020, Gomes et al. 2021), and speculating on the recovery and future of international education (Hurley, 2020). Meanwhile others use their research to provide strategies to help international students during the pandemic (e.g. Gamlen, 2020) and effective ways to engage with international students in the online space (Gomes, 2017). What the research has *not* addressed, however, is whether higher education student support services are able to keep up with the evolving international student experience and, thus, provide suitable wellbeing support to match students’ needs. The scale and impact of COVID-19 on international students are unmatched with no historical template to understand if higher education student support services truly understand the lived experiences of students whose education, expectations, aspirations and daily lives have been left with unending uncertainties. COVID-19 has placed student wellbeing as central to the international student experience.

While COVID-19 presents uncertainties around the landscape of international students and their mobility at the time of data collection in 2021, it does capture the unpredictable lived experiences of university-going international students and the ability of higher education student support services’ approaches and strategies to meet the wellbeing needs and expectations of these and future international students in a rapidly changing world. By focusing on HDR international students studying face to face or remotely in Australia and overseas, this paper captures the experiences of students undertaking Australian education and assist higher education student support services to respond to the wellbeing needs of students whose international education experiences are forged by the evolving (and lingering) impacts of a global crisis.

## Introducing shock temporality

To understand the complexities and nuances of international student challenges and behaviours as they deal with the uncertainties caused by the pandemic and its aftermath, it is necessary to construct an interdisciplinary theoretical framework drawn from cultural studies, mobility studies, migration studies and cognate disciplines Specifically, these include Xiang and Sørensen’s (2020) emerging conceptualisation of shock mobilities where acute disruptions affect human activities; and Greene et al.’s (2002) theory of resilience through positive coping, adaption and persistence. This paper builds on my theorisations that temporary migrants create parallel societies while overseas (Gomes, 2015, 2022) and the concept of ‘transience as a method’ (Gomes, 2019) which points to the uncertainties, complexities, nuances, behaviours and ecologies which emerge from the migration experience. This paper also builds on research on technology, mobility and international students’ social media use (Gomes, 2017). Here work points to the digital space as crucial in a) providing transnationally mobile subjects support through the enabling of ongoing connections with social and family networks, b) seeking information and c) wellbeing (Gomes, 2022).

This paper, therefore, advances concepts of wellbeing by paying attention to transience, adaptability and agency by building on the work done by Sawir, et al. (2008) and contributes to emerging research on international students including theoretical work on international student and digital engagements (Gomes, 2017). This paper, thus, elicits from HDR international students how they are adjusting to new (post)pandemic challenges, thus, creating conceptual understandings of students in crisis which will inform new ways of interpreting the experiences of international students through COVID-19 and recovery.

To this end, this paper introduces the term ‘shock temporality’—affecting not only this study’s participants’ professional but also their personal futures.^3^ Shock temporality is one where the challenges posed by a sustained crisis leaves a transient migrant’s (e.g. international students)  professional and personal agendas and aspirations in suspension due to sudden and extended temporality. The expected and finite temporary/transitory experience becomes forcefully broken and appears to be ongoing due to external factors outside of the individual’s control. This results in the expectations and consequences of the transient migrant’s journey becoming disrupted where the transient experience stops being linear. Individuals, thus, are unable to move on and progress in fulfilling their agendas and aspirations of which transience is the precursor. Shock temporality leads to transient migrants confronted with uncertain, precarious and unfulfilled futures with individuals unable to move from their current point of time. Moreover, this current point of time becomes endless and may seem monotonous.

This paper, thus, reports on the impact of shock temporality caused by the global COVID-19 pandemic on 8 current and recently graduated higher degree by research (HDR) international students from public institutions in the Australian city of Melbourne.^4^ The reason for this paper’s investigation of HDR international students is because international education stakeholders (e.g. governments and institutions) as well as higher education commentators have largely ‘focused on the loss of income from undergraduate international student enrolments curtailed by cross border bans and restrictions’ (Gomes et al. 2021). The voices of HDR students, however, have been marginalised and so have concerns on their wellbeing due to lower numbers of HDR international who make up less than a third of international students in Australia. Moreover, HDR international students, often on Australian government or institutional scholarships, are not as directly impactful on the Australian economy as full-fee paying undergraduate and diploma international students (Gomes et al. 2021, p. 20).

Participants, as reported in this paper, reflect how their planned professional and personal futures have become suspended due to the rolling impacts of COVID-19 on their professional and personal lives, and aspirations. While participants report that the trajectories of their professional and personal futures are in limbo or on hold, they also reveal that they are developing coping strategies as they adapt and rethink their disrupted lives and aspirations during the pandemic-induced sudden and extended temporality.

## The impact of the pandemic on HDR international students in Australia

The lived experiences of international students have been disrupted and are in a state of flux. In their small study of the experiences of HDR students during the COVID-19 pandemic, for instance, Gomes et al., (2021, 26-27) summarise the fallout of closed borders and lockdowns on the aspirations of students:

Many [graduates] are in precarious employment circumstances, such as casual retail and hospitality work, or casual university teaching, which have been significantly impacted by the imposition of pandemic restrictions and related cost-cutting measures. This precariousness has impacted on the wellbeing of HDR students in several ways. For some, the loss of a job or reduction in hours had resulted in heightened anxiety about their ability to support themselves and their families …. Other students felt that the university supported them as HDR students, but as casual tutors they were treated disrespectfully and with a lack of concern, such as being informed via email that contracts would be reduced or not renewed with little or no notice…. Other students felt that the university supported them as HDR students, but as casual tutors they were treated disrespectfully and with a lack of concern, such as being informed via email that contracts would be reduced or not renewed with little or no notice.... Outside of the uncertainty and precariousness of immediate work situations and their economic and emotional impact, students were also concerned about their future career prospects, particularly in light of the Australian Government’s changes to fees and funding in the arts and humanities.

COVID-19 and the ongoing effects of the global pandemic has created a situation where higher education student support services face challenges understanding the lived of experiences of international students (Veerasamy & Ammigan, 2021) generally because of (a) lockdowns preventing students from interacting with student support professionals (Bruhn-Zass 2021) and, (b) student wellbeing needs and expectations changing due to the impact of lockdowns and border restrictions (Coffey et al. 2021). This paper’s focus on the lived experiences of international students—with a particular focus on higher degree by research students—provides student services with initial data on the coping strategies students use when faced with difficulties and challenges. This data will be useful in assisting student services create effective wellbeing approaches and strategies especially for students facing crisis..

While this paper acknowledges that international students in Australia have faced heightened and new vulnerabilities (e.g. xenophobia, and financial, food and accommodation insecurity, Farbenblum & Berg, 2020) it also acknowledges that international students are discovering new forms of adaptability (e.g. forming informal support networks, Gomes, 2018) as they encounter unforeseen challenges (e.g. futures on hold) brought about by the impact and uncertainties of the COVID-19 global pandemic. While there have been in-depth studies on the off-campus lived experiences of specific groups of international students in Australia based on nationality (e.g. Martin, 2020 on female Chinese students), sector (e.g. Tran & Dempsey, 2017 on VET; Gomes, 2022 on higher education international students) and issues (e.g. Morris et al., 2020 on accommodation; Farbenblum & Berg, 2020 on labour exploitation; Gomes, 2017 on digital engagements), this paper provides a portrait of s HDR international students facing uncertain futures in what I explain as ‘shock temporality’.

## Temporalities and transience in migration and mobilities studies

This basis of this paper’s theorisation of ‘shock temporality’ builds on the growing literature on temporality in migration and mobility studies (e.g. Collins, 2012; Cresswell, 2012; Glick Schiller, 1999). This work, summarised by Baas and Yeoh (2019) in the introduction of their edited collection on time and temporality, tell us that scholarship on temporality is based on ‘the way migrants across the skills spectrum experience, negotiate and engage with the various temporal aspects of their trajectories, especially in relation to structural constraints and opportunities’ (p. 164). This journey, however, is fraught with twists and turns which are primarily governed by the visa policies of destination countries (Baas & Yeoh, 2019). Here Baas and Yeoh (2019) explain that there are aspirational migrants who become stuck as immobile subjects, unable to move transnationally due to lack of skills required by destination countries; thus, leading to an inevitable wait time in the path of mobility and eventual settlement. It is during this wait time that individuals may invest in training (or retraining) to gather the skills necessary for the end goal of settlement (Soong, 2015). In other words, the act of temporality or transience for international students is one that is aspirational where people enrol in education programmes overseas so as to create better futures for themselves whether in the host country, the home country or elsewhere.

This paper also builds on the ample empirical evidence pointing to transience as a lived migrant experience such as my previous work (Gomes, 2019) advocating the use of transience as a conceptual lens to decipher transnational migrations. Here I discuss temporary migrants (international students, working holiday makers and university-educated professional workers) in the Asia–Pacific and suggest that the concept of transience ‘challenges what we understand about cross border migration and should be used as a method in comprehending the complexities, nuances and ecologies emerging from these migration experiences which then impact on emerging patterns of (ethnic and cultural) diversity’ (2019: 2). Interviewing 277 individuals and surveying 7,084 others in online surveys from 3 different projects over 5 years, I note that not only are transnational migrations becoming increasingly transient but also the concept of transience has become common with migrant(ion)s evolving into sophisticated actors/actions requiring attention. I explain that the idea of transience is becoming more significant since ‘it defuses permanence (in permanent migration) as well as subverts the temporariness in temporary migration’.

However, the COVID-19 pandemic is a completely new variable severely affecting governmental policies on mobility whether they affect neighbourhood, intrastate, interstate or international travel. Such measures to slow down the spread of COVID-19, however, have resulted in uncertainties and precarities never before seen. So while the literature on temporalities does indeed recognise that there are ‘twists and turns’ (Baas & Yeoh, 2019) in the migration journey where there is a desire for temporality to lead to permanence of some kind, and others (e.g. Gomes, 2019) note that even permanent migration can be transient, the pandemic has added a new dimension to the conceptualisations around temporalities and transience. Here the suddenness of unplanned temporality inevitably provides a ‘shock’ to the temporary/transient migrant experience.

## Coping with shock

In 2020, anthropologist Biao Xiang coined the term ‘shock mobilities’ to explain ‘sudden human movements made in response to acute disruptions, such as the present COVID-19 pandemic (Xiang, 2020). Explaining the concept further, Xiang together with Ninna Nyberg Sørensen (2020) writes

Unlike planned migration, shock mobility encompasses various degrees of forced migration or can be categorized as reactive migration caused by a crisis situation …

Here Biao and Sørensen rightly observe that during times of crisis, people are forced to suddenly move as a response to external factors out of their control. When migration—whether interstate or international—is not planned, mobile subjects have to deal with the practical and emotional aspects of this sudden movement. This is because the process of migration is complex in its practicalities requiring logistical planning not only *from* a place but *to* a place. For instance, properties and belongings need to be sold and/or relocated. Migration is also an emotional process where migrants deal with the stress and feelings of leaving the familiarities of place and people from the moment they make the decision to leave. In Australia, international students faced shock mobility in the early months of the pandemic in 2020. Leaving Australia abruptly to return to their home countries to wait out the pandemic, international students continued with their studies remotely as education institutions migrated all teaching and learning online. News reports and social media (Lehmann and Sriram, 2020) at the time showed images of the frontage of rental properties once occupied by international students littered with their abandoned furniture. International students suddenly left Australia due to home country governments recalling them back to be with their families during the pandemic. Conversely, Xiang (2020) also recognises that with shock mobilities, there is also shock immobilities. He explains

First, urban middle class isolated themselves because they were sensitive to health risks, and can afford to stay at home. And second, rural communities were quick in setting up checkpoints and even building walls around villages because, due to poor healthcare facilities, physical isolation appeared to be the only thing that they could do to protect themselves.

In other words, shock (im)mobility leads to people being cut off and unable to move beyond their home (e.g. village, city, state or country). Likewise, transient migrants who are stuck and unable to move beyond state and national borders where they find themselves isolated. ‘Shock temporality’, thus, differentiates itself from ‘shock (im)mobilities by expanding on the experience of the transient migrant who though immobile is also lengthening their temporary state. So, unlike the citizenry who are ‘at home’, transient migrants are expecting their temporality to cease at a certain (and designated) point in time rather than to continue with no end in sight. Recognising the concerns around international student (im)mobility, a growing number of scholars (e.g. Ferdiansyah, Supiastutik and Angin, 2020; Farbenblum & Berg, 2020) have been researching into the disruptions caused to international student mobility in terms of disrupted and halted professional and personal aspirations. While scholars such as Xiang and others are responding to the new (im)mobility experiences of international students, scholarship on the impact of the COVID-19 pandemic on their everyday lives and futures is understandably still at an early stage. Research, however, emerging at the time this paper was being written, has concentrated mainly on potential international students whose mobilities and, hence, aspirations for overseas study and migration being curtailed (Cheng, 2020), the challenges international students face in terms of study (Gomes, 2021) and concern around their wellbeing (Humphrey & Forbes-Mewett, 2021), with little on international student coping strategies in the face of what seems to be lives and futures in limbo.

## Method

This pilot project is dedicated to improving the wellbeing of international students by comprehensively understanding their lived experiences. Here this project consults international students studying in classrooms as well as remotely within Australia and overseas so as to provide initial data and recommendations to student support services in creating solutions for improved student wellbeing. Ethics approval was granted in late 2020 while data for this pilot study was collected from March to June 2021 via Microsoft Teams with each interview lasting around 30 min. There were several reasons why interviews took place online: Melbourne was in lockdown in various periods during data collection; participants were residing interstate and overseas; and the ease of conducting interviews online. For the latter, speaking to participants online meant saving time and money on travel particularly since they were not compensated for taking part in this research. Moreover, interviews, regardless of where participants were located, could be scheduled at their convenience.

Eight people participated in this project. Demographics of participants are found in Table 1.

**Table 1:** Demographics of participants

Pseudonym	Level of study	Region/Country of origin
James	Recent PhD graduate	Africa/Ghana
Zack	Recent PhD graduate	Northeast Asia/China
Aaron	Recent Masters graduate	Northeast Asia/China
Alice	Recent Hons graduate	Mauritius
Hsien	Current PhD student	Southeast Asia/Malaysia
Olivia	Current Masters student	Southeast Asia/Singapore/Burma
Sharon	Current PhD student	Northeast Asia/Hong Kong
Caron	Current PhD student	South America/Columbia

As Table 1 shows, four of the participants recently graduated from graduate studies (2 PhD, 1 Masters and 1 Hons) and four are current graduate students (3 PhD and 1 Masters).

Recruitment for participants took place through advertisements in newsletters such as the City of Melbourne newsletter, through social media such as LinkedIn and Twitter, and through snowballing. When interviewing participants, the following themes were transferred into the chat section of Microsoft Teams for participants to refer to during the interviews:

a. Accessibility to goods and services—e.g., food security, accommodation, digital accessibility [platforms, services and content], access to healthcare, personal safety.

b. Employment/Employability—e.g., during studies and for the future.

c. Social and cultural cohesion—e.g., social networks, relationships and community, sense of belonging, emotional and mental wellbeing, religious and cultural freedoms and expressions, cultural shocks, geopolitics, racism, xenophobia.

d. Self-perceived identities—e.g., genders, sexualities, nationalities, ethnic cultures.

e. Aspirations—e.g., pre and post study agendas and ambitions.

f. Media and communication use—e.g., entertainment, news and information-seeking.

These themes are drawn from the work of scholars on international student wellbeing in Australia (Farbenblum & Berg, 2020; Morris et al., 2020; Forbes-Mewett, 2019, Gomes, 2022) which are connected to international students’ off-campus experiences. Participants were then asked to provide a reflection of the pandemic’s impact on them based on the following questions in reference to the themes:

1. What are the challenges you face navigating the digital and physical off-campus environments, even if you are studying in your home country (e.g., digital accessibility and overcrowded households)?

2. What are the strategies and approaches you use to deal with and/or adapt to the challenges you encounter?

3. What are the challenges you are unable to handle or find difficult to handle?

While originally this project wanted participants to reflect on 2020, it became apparent as participants spoke, that their experiences and concerns were still ongoing. These included challenges caused by the COVID-19 pandemic—e.g,. closed international borders preventing participants studying remotely in their home countries from returning to Australia; closed international borders preventing participants in Australia from visiting the home country; closed international borders preventing participants travelling elsewhere even for post-graduation work for fear of not being able to return to Australia.

Sometimes some of these challenges intersect. For instance, fresh PhD graduates felt that the pandemic made getting academic positions in an already hard-to-get-into education sector challenging. The findings of this study reveal that participants were experiencing suspended professional and personal futures due to their state of shock temporality. However, this paper also reports on how participants are using the time of sudden and extended temporality to make and action concrete plans aimed at building up their professional and personal futures (e.g., doing volunteer work to build their resumes for future work and exploring legal avenues to be reunited with romantic partners).

## Findings

### Shock temporality suspended futures

#### Professional lives in limbo

Since 2020, there have, unsurprisingly and necessarily, been snap studies intending to understand how tertiary-going students cope during the pandemic. Surveying 1200 PhD students at their Australian institution, Johnson et al. (2020) found that half the students wanted to give up their doctoral studies because of negative job prospects for PhD graduates in academia. Clearly the shock temporality caused by the pandemic where the sudden and extended real sense of lives in limbo have clear implications for HDR (post) graduate futures. Participants in this study, for instance, discuss their suspended aspirations where they articulate that their professional (academic) and personal lives are on hold. Zack a recent PhD graduate, for instance, felt the challenge of shock temporality where he considered his life ‘wasted’ due to a real feeling  he was suspended and in limbo. However, while participants felt a sense of despondence over their current situation—as Xiang and Sørensen say, ‘wasting time’—they were in actuality being proactive in building their professional and personal futures. Moreover, participants had been using their extended temporality for self-improvement and self-discovery.

Unlike previous studies on the impact of the pandemic (e.g. Johnson et al., 2020) where employment was not an expressed concern for current HDR students, employment was a key issue for participants in this study. PhD graduates, in particular, said that they were finding difficulty getting jobs in Australia. This was not only because of the scarcity of employment in academia but also because of their temporary visa status. James, a recent PhD graduate for instance, felt that it was hard to get full-time employment as an international student in Australia. He felt ‘neglected [by Australia…. in [my] moment of need’ because he was not a citizen or a permanent resident. He further explained:

Will I ever get a job in academia? [W]ould I look good enough to get a job in government sector? [W]ould I even be considered—taking into consideration that my visa is you know [is an international student]. There's also unwritten rule of employees being citizens or PR people. [This is] something that I worry about but I'm a very positive person and I try to make the best out of even the [worst situation]. So, what I told myself that …. perhaps in the next two or one year or three years the economy will pick up so {university] schools [might] want to employ more people. I'll be marketable or …. competitive enough [by then] …..I'm working on publishing so that if somebody asks me ‘so 2021 what do you with your time since you do not have a full time job?’ [So, I will] concentrate to publish so that I bring that kind of experience.

Similarly, Zack, also a recent PhD graduate, felt that the impact of COVID-19 had disrupted his career plans (e.g., applying for postdoctoral positions overseas before returning to Australia). He believed that the pandemic hit PhD graduates—especially international PhD graduates—badly in terms of their choices for employability especially in academia. He explained:

Now it's about uncertainty. Obviously for international students like myself, for transient migrants, uncertainty or precarity is always is a thing there. But I guess what adds to that kind of existing degree of certainty is what the pandemic brings about, right? The weather you are not sure whether your decision is good or not is the correct one or not so.

Zack dealt with this employment uncertainty and precarity in an already precarious sector by continuing to publish from his doctoral research. However, Zack had also been strategic in creating both a professional and personal future for himself. He explained that he had given up casual teaching in favour of a research-intensive casual positions due to eligibility requirements for permanent residency in Australia. This is because permanent residency applications for PhD graduates at the time when we spoke favoured a track record in research activities for early career researchers (Australian Government, Department of Home Affairs, 2021c). Hence, Zack spent his time publishing from his doctoral thesis and working in casual research positions as building blocks for his future in Australia and in academia. Such a strategy for Zack is understandable since citizens and permanent residents are favoured over temporary migrants for permanent full-time employment in Australia.

Alice, who graduated at the end of 2020 with an honours degree, felt that employment post-study was an all-consuming issue for her:

Yeah, but I did feel a bit of pressure as well when it comes to employment and it's already because it was my final year in 2020.

Alice explained that she sent out over 100 job applications in 2020. However, she persevered applying for jobs and, in conjunction, attended online networking events as building blocks for future work. Throughout the pandemic, online networking events dedicated to introducing current international students and fresh international student graduates to potential employers together with online workshops with topics to enhance employability such as resume writing and interviewer techniques were made available free of charge. These were designed by state governments (e.g., the State Government of Victoria), local councils (e.g., City of Melbourne), university institutions and non-government organisations (e.g., VicWISE who advocate for international student welfare).

#### Personal life on pause

For some of the participants such as James (recent PhD graduate) and Olivia (current Master’s student), the closed borders had severely affected their personal lives. When we spoke, James had recently entered into a romantic relationship, but his partner had moved interstate to Sydney for reasons he did not disclose. Hence, James was experiencing a long-distance relationship via Zoom and wondering how other people maintained such relationships. While James’ relationship was with someone still in Australia, Olivia, who relocated back to Singapore when the pandemic began to be with her family, was maintaining a transnational long-distance relationship.

For Olivia, maintaining a long-distance relationship was the most difficult part of the pandemic. While still in Melbourne, Olivia was living with her boyfriend who she admitted, she missed tremendously. While the relationship was more challenging earlier in the pandemic, it had started to improve due to what she felt was her continuing maturity. To deal with maintaining a long-distance relationship, Olivia joined a private Facebook group called ‘Partners Apart’ to cope but found that membership was too emotional since everyone ‘pours their issues onto the site’.^5^ Hence, Olivia now only looked at certain posts which she wants to read and avoids the rest. Furthermore, she and her boyfriend had taken steps to be together by seeing an immigration lawyer. Olivia also registered her relationship with the Victorian State Government’s Registry of Births Deaths and Marriages (2021) as proof that they have a legitimate relationship when she eventually applies for permanent residency.^6^

Olivia informed me that she felt that it was more practical for her to live permanently with her boyfriend in Australia despite him agreeable to move to Singapore to be with her for a couple of reasons. First, Singapore, unlike Australia, does not accept de facto relationships; thus, her boyfriend would have to find a job for himself in order to live with her. Moreover, he would have to secure a skilled work visa matching his qualifications and earning power (Ministry of Manpower, Singapore, 2021). Second, Olivia’s boyfriend has personal responsibilities and ties in Australia. He recently purchased a house in Australia with a mortgage to pay. He also has elderly grandparents in their 90s whom he is close to. Olivia was of the opinion that her boyfriend would feel tremendous guilt if he was to relocate to Singapore—an emotion she did not wish upon him.  

### Shock temporality: making the most of sudden and extended temporality

Emerging research on international students in Australia (e.g. Farbenblum & Berg, 2020; Lehmann & Sririam, 2020) and elsewhere (e.g. Yu, 2021) across the higher education sector reveal that lockdowns and international travel restrictions have impacted their mental wellbeing and aspirations similar to those reported by participants in this paper. While it is important to discuss the challenges international students and recent graduates in the higher degree space experience during the COVID-19 pandemic, it is equally important to understand how they cope with and adapt when they feel their lives and futures are in sudden and extended temporality. With no previous understanding of how HDR international students navigate interrupted life courses and uncertain futures during a worldwide crisis, how do students cope with the challenges of precarity during an unpredictable global pandemic? Participants in this study explained that they needed to concentrate on taking care of themselves (e.g,. their own wellbeing) since they were unable to do anything to change the state of the world. Participants, in other words, felt that they had to see the positives rather than dwell on the negatives (Greene, 2002; Ploner, 2017) as a way of dealing with a situation for which they had no power over.

For instance, Alice realised that to get through the uncertainties of the pandemic, she would have to tell herself that she should concentrate on what she can control such as keeping in touch with friends and family, and finding community with her honours cohort while letting go of what she could not control such as worrying about closed borders. She revealed that she got through feelings of helplessness during the pandemic by telling herself that others were ‘in the same boat as her’. Hence, she convinced herself that she was not alone in this regard. Alice also told herself not to dwell on what she was unable to change (such as the pandemic) and to be kind to herself. She, thus, resorted to watching a lot of videos online to take her mind off the COVID-19 crisis. Participants moreover reported that they continued to use the time afforded to them by shock temporality to build up competencies for future work as well as to develop new skills.

#### Building blocks for the future

Participants who were finishing their degree or graduating in 2020 were unsurprisingly working towards future employment. Non-PhD student and recently graduated participants spent their time upskilling (e.g,. taking part in online workshops which helped them write better resumes and improve their interviewee techniques; and attending online networking events organised by their institutions and by international education stakeholders) to improve their chances at getting a job. For instance, Aaron who graduated at the end of his Master’s course felt that he ‘need[ed] to do something more meaningful’ so he devised a detailed plan to make himself employable. He felt that he ‘need[ed] to change [his] mindset from closed to open’. So, he enrolled in programmes outside of his degree course to improve his chances at employment. He also researched the organisations he was applying jobs to and was careful about editing his communication (e.g., resumes and cover letters) to prospective employers.

Meanwhile graduating PhD students who were concerned about entering academia and, thus, committed to increasing their publication record in the hopes of securing postdoctoral employment. Dealing with the impact the pandemic was having on his career trajectory, Zack revealed that he did not want to make sudden decisions about his future. Instead, as noted earlier in this paper, he felt that he needed to continue working on building his career by taking on casual research assistant jobs and publishing from his doctoral research. He rationalised his decision-making processes as linked to his cultural upbringing in China:

In Chinese, there is this kind of saying. Well, let me try to translate it right. It is about carrying on and not making rash decisions, but rather to ride out the situation just in case the rash/sudden decisions might be the wrong ones. Well, hopefully the translation makes sense, but I guess that's kind of the way of dealing with crisis of dealing with our sudden precarity. That is, you don't want to just make changes when there's a change out there, but you want to make sure that you have observed everything and you kind of considered everything before you make that decision.

Investing time in their employment futures, however, was not the only pursuit on participants’ minds.

### New pursuits facilitated by communication technologies

Discovering and then pursuing their new talents through communication technologies afforded to them, helped participants through the pandemic. Current PhD student Caron, for instance, took up watercolouring during the pandemic and found that it calmed her; so much so that she watercoloured while we spoke. This is because Caron realised that talking about the impact the pandemic had on her would be distressing. Caron explained that water-colouring moreover allowed her to make friends with new people and to reconnect with existing friends. Hence, Caron regularly met up with these friends online to watercolour:

I decided to like when I started the watercolours. I always wanted to watercolour with friends, and I never found anyone in Melbourne who wanted to do it and finally I found someone. Then came locked down so we decided OK let us see [doing] it every day….[S]o it was just the two of us like for three months and then I remember a friend that I met like 10 years ago or even more when I was in Holland doing my Masters….I knew she really likes water colouring so I also texted her [if she wanted to] watercolour with us. So we then [asked] …. another friend in Melbourne to also join but mainly [it is] the three of us

James meanwhile found purpose in disc jockeying (DJing). He felt that this newly discovered talent provided him with an avenue to contribute to community. James, who is from Ghana, had been active in organising events for HDR students from the African community in Melbourne prior to the COVID-19 pandemic. Since the pandemic, James had taken to using the online platform Zoom for such get-togethers which he revealed was more popular than the face-to-face events. It was during these events that he played the part of DJ where he featured music from the different African countries represented by the students in attendance. James rationalised that the increased attendance in the online space was an indication that people needed to feel connected to others who they identified with in terms of what he felt were ‘similar values'. Here he indicated that one such value was a concern for fellow Africans who were facing racial discrimination in China—an issue which had become more intense during the pandemic (Human Rights Watch, 2020).

## Discussion and conclusion

The disruptive effects of the COVID-19 pandemic and its unknown, evolving and diverse long-term impacts on current and future international students have severely impacted the ways higher education student support services service international students undertaking study onshore and remotely overseas. In particular, higher education student support services face current and future challenges when creating suitable wellbeing approaches and strategies for international students in the key areas of their responsibility (Veerasamy & Ammigan, 2021). Specifically, in Australia these areas are: (i) health, safety and wellness (including counselling services, chaplaincy and LGBITQ + support); (ii) careers and employability advice (while undertaking study and in preparation for entering the job market post-graduation); and (iii) financial and legal support (while enrolled). This paper, thus, set out to understand the higher degree by research international students to assist higher education student support services create approaches and strategies that service Australian international students in these three key areas by understanding their lived experiences and expectations which have pivoted because of the impacts of the pandemic (Coffey et al. 2021). While the pandemic heralded temporary hardships (e.g. job losses), restrictions (e.g. due to lockdowns) and stresses (e.g. isolation), it has highlighted wellbeing as a key issue for international students with fundamental long-term implications on international education. The international education sector in Australia (and elsewhere), thus, must prepare for a future where effective student support outside of curriculum and pedagogy becomes critical to student recruitment and retention with the higher degree by research space used as the example in this paper.

The shock temporality caused by the COVID-19 pandemic’s halt to local and international mobility due to lockdowns and closed state and national borders have left international students interviewed in a state of uncertainty and precarity as they felt and experiences their lives left in limbo. The current and recently graduated higher degree by research (HDR) international students interviewed for this study revealed that they saw their professional and personal futures on pause. Participants, however, had been making the most of shock temporality where the fear of ‘what’s next’ after graduation had made them proactively work towards creating better professional and personal futures. Two clear interrelated themes thus emerge from this pilot study: (1) the increased and diverse use of information and communication technologies to contend with challenges in career and intimate relations; (2) the exacerbated effect of a precarious migration status on both career and intimate relations. These themes resulting from shock temporality assist institutions to understand HDR international students’ non-study experiences of the (post)pandemic so as to create effective wellbeing approaches and strategies for these and future students.

While this paper reports on shock temporality on a small group of current and recently graduated HDR international students, would the findings be similar or different with students working towards undergraduate, diploma and/or certificate qualifications? This is because HDR students and recent HDR graduates are often older than undergraduate (and possibly diploma and certificate) students and, hence, assumed to be more mature in terms of life and work goals. For the latter, students who enrol in doctoral programmes—which are specialist research training degrees—primarily because of a desire to enter the research community whether this may be in academia or in industry. A PhD, for instance, is a requirement to enter academia (e.g,. as a teaching and research academic) in most universities worldwide. This paper thus observes the following needed by higher education providers to create effective international student wellbeing approaches and strategies:

(i) new knowledge on the international students lived experience during crisis;
(ii) new knowledge on how international students cope with challenges and difficulties;
(iii) new knowledge on the emerging lived experiences of international students as a result of living in a (post)pandemic world.

This paper, therefore, suggests that higher education institutions consider developing employability programmes that support HDR students—both international and domestic—at the end of their graduate degrees and after graduation. While institutions as well as international education stakeholders (e.g., government and non-governmental organisations) run workshops to assist international students with improving their employability skills (e.g,. resume writing and interview techniques), HDR (international) students require more specialised assistance. Such specialised HDR programmes, for instance, might assist current students about employment options in both academia and industry in the host country and overseas. Programmes might also include job and grant application training, and mental wellbeing support to cope with the employment realities of both HDR students and early career researchers. While institutions may run some of such programmes for their current HDR students, they should do so for their recent graduates as part of alumni engagement regardless of whether they are located in the host country or elsewhere.

Shock temporality, thus, allows for an understanding of what happens when people are ‘stuck’ in a temporary or transient migration situation which they understood to have a finite lifetime. Instead because of factors out of their control, being ‘stuck’ in what seems to be never-ending temporality leaves lives, aspirations and futures suspended. Shock temporality, in other words, brings a new dimension to the temporalities literature and provides a useful framework in understanding a sudden halt to linear temporal temporality. Shock temporality, thus, becomes a valuable concept in understanding the temporary migrant experience. In the case of international students who are temporary/transient migrants, shock temporality becomes a useful tool for student services to understand international students in crisis.

The COVID-19 pandemic, therefore, provides migration and mobility researchers and international education stakeholders charged with international student wellbeing comprehend what international students  go through during shock temporality where lives, aspirations and futures are suspended. Using the shock temporality framework, this paper provides higher education student services with a tool to understand not only the international student experience during a crisis but also how to help students. While this paper looked specifically at the higher degree by research (HDR) international student, it provides an entry into how higher education student services can assist not only HDR international students but international students more broadly during times of crisis.

## Footnotes

1 Higher degree by research (HDR) students are also known outside of Australia as graduate students and postgraduate students.

2 As I was working on this paper, the Department of Anthropology and Sociology at the University of Western Australia – a G8 university – was in the midst of being dissolved by university management in order to make savings of AUD40 million resulting in 16 academic staff being retrenched (Styles, 2021).

3 Transient migrants are migrants who are temporarily in a country for a limited amount of time for specific and sometimes intersecting study, work, humanitarian and lifestyle reasons. For instance, international students choose countries to study in because they want to experience the lifestyle offered by the host country. Transient migrants not only spend limited time in the host countries but have limitations concerning their rights. In the case of international students in Australia, they are provided with visas which cover the course of their study and have to return home or apply for another visa (e.g., for further study or work) once they receive their academic credentials. They also do not have the same welfare benefits and work rights as Australian citizens and permanent residents. During COVID-19, international students were ineligible to receive federal government financial aid. Instead, state governments and institutions made available financial aid for students through applications of hardship. Likewise, non-government organisations organised food banks for students in need. While international students in Australia are only allowed to work for 40 hours per fortnight, the federal government eased these restrictions in order for students to fill employment gaps in certain industries such as age care, tourism, agriculture and retail particularly supermarkets (Australian Government, Department of Home Affairs, 2021b).

4 Australian higher education is divided into state and private ‘for profit’ institutions where the latter is based on a business model primarily rooted in recruiting and educating international students.

5 According to its Facebook description, Partners Apart is a ‘[d]iscussion & support group for people in Australia & their partners being held apart due to the current world situation and global travel bans’.

6 In Australia, individuals can apply for permanent residency through a partner visa which allows the person to stay in Australia first on a temporary basis and then long term as a permanent resident (Australian Government, Department of Home Affairs, 2021b).
